# Multifocal VEP and OCT findings in patients with primary open angle glaucoma: A cross-sectional study

**DOI:** 10.1186/1471-2415-12-34

**Published:** 2012-08-02

**Authors:** Marilita M Moschos, Gerasimos Georgopoulos, Irini P Chatziralli, Chryssanthi Koutsandrea

**Affiliations:** 1 Department of Ophthalmology, University of Athens, 6, Ikarias street, Ekali, 14578, Athens, Greece

## Abstract

**Bakground:**

To evaluate objectively the anatomical and functional changes of optic nerve in eyes with primary open angle glaucoma (POAG) by the joint use of optical coherence tomography (OCT) and multifocal visual evoked potentials (mfVEP).

**Methods:**

29 eyes with open angle glaucoma and visual field defects, as well as 20 eyes of 10 age-matched control normal subjects were tested. All participants underwent a complete ophthalmological examination. Moreover, Humphrey visual field test, OCT examination and recording of mfVEP were performed. Amplitude and implicit time of mfVEP, as well as RNFL thickness were measured. Differences in density components of mfVEP and in RNFL thickness among POAG eyes and control eyes were examined using Student’s t-test.

**Results:**

In glaucomatous eyes the mean Retinal Response Density (RRD) was lower than normal in ring 1, 2 and 3 of mfVEP (p < 0.0001). Specifically the mean amplitude of mfVEP in POAG eyes was estimated at 34.2 ± 17.6 nV/deg^2^, 6.9 ± 4.8 nV/deg^2^ and 2.6 ± 1.6 nV/deg^2^ in rings 1, 2 and 3 respectively. In contrast the mean implicit time was similar to control eyes. In addition, the mean RNFL thickness in POAG eyes was estimated at 76.8 ± 26.6 μm in the superior area, 52.1 ± 16.3 μm in the temporal area, 75.9 ± 32.5 μm in the inferior area and 58.6 ± 19.4 μm in the nasal area. There was a statistically significant difference in RNFL thickness in all peripapillary areas (p < 0.0001) between POAG eyes and controls, with superior and inferior area to present the highest decrease.

**Conclusions:**

Our study shows that, although Standard Automatic Perimetry is the gold standard to evaluate glaucomatous neuropathy, the joint use of mfVEP and OCT could be useful in better monitoring glaucoma progression.

## Background

Glaucoma affects over 70 million people worldwide and is considered to be the second most frequent cause of blindness [1,2]. Although the central visual fields and visual acuity are preserved until the late stages of glaucoma, in many cases visual acuity is decreased even in the early stages of glaucoma [3,4]. Indeed, it is estimated that a loss of 20% of retinal ganglion cells (RGCs) is necessary to detect a 5 dB decrease in mean deviation (MD) of the standard automatic perimetry (SAP) [5]. Therefore, examination by SAP is not always adequate for early diagnosis and monitoring of glaucoma.

On the other hand, optical coherence tomography (OCT) is a high resolution non-invasive method which can quantify the optic disc and the circumpapillary retinal nerve fiber layer (RNFL) [6,7]. In addition, multifocal visual evoked potentials (mfVEP) is one of the recently used tests that may objectively assess visual function and detect glaucomatous damage. Different studies report that mfVEP as well as OCT can detect and monitor visual field defects more accurately than SAP, which depends on the subject’s response criterion [8-10]. In light of the above, the purpose of this study is to evaluate the joint use of OCT and mfVEP in the assessment of the structural and functional changes of optic nerve in eyes with primary open angle glaucoma (POAG) and visual field defects.

## Methods

Patients with POAG [11] were recruited from the Department of Ophthalmology of Athens University (Glaucoma Unit). Informed consent for imaging and data collection was obtained from all patients after explanation of the nature of the study. The study was conducted in accordance with the tenets of the Declaration of Helsinki and was approved by the Institutional Review Board of “G.Gennimatas” General Hospital of Athens, Greece.

Inclusion criteria were any of the following: POAG with glaucomatous optic neuropathy in at least one eye, defined as a cup-disc ratio ≥ 0.6 on fundoscopy by two independent examiners, and repeatable reliable visual field defect on SAP. Specifically, all patients had a Schaffer III-IV angle in gonioscopy and presented a typical glaucomatous visual field defect i.e., Bjerrum, altitudinal or nasal step scotoma. Additionally, the intraocular pressure was under control with topical medication treatment (17.1 ± 2.3 mm Hg). The patients had no history of other ocular disease or eye surgery. Twenty two patients with a total of 29 eyes affected with POAG were enrolled in the study (Group A). Additionally, 20 eyes of 10 age-matched patients, without ocular or systemic symptoms, participated in the study as normal control subjects (Group B). Best corrected visual acuity (BCVA) was equal to 1.0 (standard Snellen chart) in both groups. Of note, all patients had a spherical refraction less than ±3.0 D and a cylinder correction less than ±1.5 D.

All participants underwent complete ophthalmic examination, including BCVA assessment with standard Snellen chart, color vision testing by Ishihara plates, intraocular pressure measurement by Goldmann applanation tonometry, visual fields perimetry with Humphrey visual field analyser, fundus examination, OCT scan and mfVEP recording.

### Visual field test (VF)

We performed VF test twice for each patient, with an interval of 30 minutes for the patient to rest. We used Humphrey 24–2 SITA testing algorithm, 54 individual points were tested, and the threshold value calculated for that point was compared to a database of normally-sighted individuals of similar age. Based on this comparison, the value for the threshold value at this location was classified as being normal, or abnormal at a 5%, 2%, 1% or 0.5% probability. Each individual location has a calculated deviation from the expected threshold value for a person of the same age and ethnicity.

### Optical coherence tomography (OCT)

OCT examination was performed with the Stratus OCT 3000 (Carl Zeiss Meditec, Dublin, California). The subjects were asked to gaze at the fixation light within the machine and the foveolar fixation was confirmed by observing the retinal through the infrared monitoring camera.

In order to measure RNFL thickness, the model uses a light emitting diode producing low coherence infrared illumination (820 nm) that generates cross sectional images of the retina with an axial resolution of less than 10 microns. For each A-scan, the OCT acquires a fixed number of 1,024 axial data points along the 2 mm depth. The total number of A-scans is 512/B-scan done in 1.28 seconds.

Retinal nerve fiber layer is differentiated from other retinal layers using a threshold algorithm that detects the separation between the first highly reflective layer (anterior edge of the internal limiting membrane) and the posterior edge the first highly reflective layer (anterior edge of the RNFL). The “RNFL thickness (3.4)” protocol is designed to acquire three circle scans of diameter of 3.4 mm around the optic disk. Good quality scans were defined as having a signal strength of ≥ 7 (maximum 10) in addition to uniform brightness across the scan circumference and being well centred at the optic nerve head. During the examination, the operator ensured exact motion-free centration.

Measurements of RNFL thickness from 3 scans were averaged to provide a mean measurement of the RNFL thickness average, as well as the following retinal regions: temporal (316° to 45° on a unit circle), superior (46° to 135°), nasal (136° to 225°) and inferior (226° to 315°).

### Recording of multifocal visual evoked potentials (mfVEP)

We used the VERIS system 4.2 (Visual Evoked Imaging System 4.2, Electrodiagnostic Imaging, San Francisco, CA). The stimulus array consisted of 60 sectors, each with 16 checks, comprising 8 black and 8 white. The stimulus array was scaled an displayed on a monochrome monitor driven at 75 Hz, The luminance of the white checks was 200 cd/m^2^ and for the black checks was 3 cd/m^2^, producing a contrast of 97%. The background luminance of the screen was 100 cd/m^2^. The borders of the rings fell at 0.5° , 3.0°, 7.0°, 12.0°, 18.0° and 25.0° retinal eccentricity respectively. To obtain mfVEP, the signals were fed into an amplifier and band-passed filtered at 3–100 Hz, The gain of the amplifier was 100,000.

For signal derivation, the active electrode was placed 2 cm above the inion and the reference electrode was placed 2 cm below the inion. A ground electrode was attached to the center of the forehead. The fellow eye was closed and the total recording time was 8 minutes.

Subjects wore appropriate refractive correction and were instructed to maintain fixation at the center of the stimulus marked with a red “X”. The colour temperature of the white of the display was 6500 k. The mfVEP waveforms were divided into five groups, from the center to the periphery, according to the different eccentricities. Because the inter-subject and intra-subject variance of traces of the outermost rings was very large, only data from rings 1, 2 and 3 were analyzed.

### Statistical analysis

Continuous data are presented as mean ± standard deviation (SD). The Gaussian distribution assumption was tested using the Kolmogorov-Smirnov test. Differences in density components of mfVEP and in RNFL thickness among POAG eyes and control eyes were examined using Student’s t-test. Pearson correlation coefficient was used in order to examine possible association between mf-VEP amplitude and RNFL thickness. The level of statistical significance was set at p = 0.05. The SAS statistical software package (Version 9.1, SAS Institute Inc, Cary, NC) was used to analyze the data.

## Results

The demographic characteristics of the study participants are summarized in Table 1.

**Table 1:** Demographic characteristics of the study participants

**Variable**	**POAG patients (n = 22)**	**Controls (n = 10)**	**p-value**
	mean ± SD	mean ± SD	
*Age (years)*	65.9 ± 9.6	60.1 ± 12.4	0.205
*Intraocular pressure (mm Hg)*	17.1 ± 2.3	14.7 ± 1.8	0.057
	N (%)	N (%)	
*Gender* Male Female	12 (54.6) 10 (45.4)	6 (60) 4 (40)	0.999

POAG = primary open-angle glaucoma.

**Figure 1:**
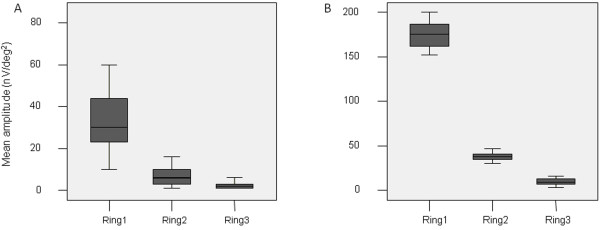
Mean retinal response density in ring 1, 2 and 3 of mf-VEP in POAG eyes (A) and control eyes (B).

Table 2 shows the retinal response density (RRD) of mfVEP, in each ring among POAG and control eyes. In all rings the mean values of RRD of mfVEP were significantly lower in the group of POAG eyes in comparison with controls. Graphical presentations of the mean values of RRD of mfVEP by area for POAG and control eyes are shown in Figure 1.

**Table 2:** **Mean retinal response density of multifocal visual evoked potentials (nV/deg **^**2 **^**) of ring 1, 2 and 3 in POAG and control eyes**

	**POAG eyes (n = 29)**	**Control eyes (n = 20)**	**p- value**
*Amplitude (nV/deg*^*2*^*)*	mean ± SD	mean ± SD	
Ring 1	34.2 ± 17.5	173.9 ± 15.0	<0.0001
Ring 2	6.9 ± 4.8	38.0 ± 5.0	<0.0001
Ring 3	2.6 ± 1.6	9.8 ± 4.1	<0.0001

POAG = primary open-angle glaucoma.

Regarding implicit time of mfVEP, as shown in Table 3, there was no statistically significant difference between the two groups in ring 1 (p = 0.869, Student’s t-test), ring 2 (p = 0.953, Student’s t-test) and ring 3 (p = 0.287, Student’s t-test).

**Table 3:** Mean implicit time of multifocal visual evoked potentials (msec) of ring 1, 2 and 3 in POAG and control eyes

	**POAG eyes (n = 29)**	**Control eyes (n = 20)**	**p- value**
*Implicit time (msec)*	mean ± SD	mean ± SD	
Ring 1	117.4 ± 24.0	116.3 ± 8.0	0.869
Ring 2	114.3 ± 20.8	114.7 ± 3.0	0.953
Ring 3	113.6 ± 24.4	106.4 ± 5.2	0.287

POAG = primary open-angle glaucoma.

Table 4 presents mean values and standard deviations of the RNFL thickness between POAG eyes and control eyes based on the type of area. In all areas, the group of POAG eyes was found to have significantly lower mean RNFL thickness in comparison to the group of controls eyes. In specific, the percentage of mean RNFL thickness decrease in POAG eyes compared to controls was 38 %, 28 %, 44 % and 25 % for superior, temporal, inferior and nasal area respectively.

**Table 4:** Retinal nerve fiber layer thickness (μm) in POAG and control eyes

	**POAG eyes (n = 29)**	**Control eyes (n = 20)**	**p- value**
*RNFL thickness (μm)*	mean ± SD	mean ± SD	
Superior area	76.8 ± 26.6	123.1 ± 6.3	<0.0001
Temporal area	52.1 ± 16.3	72.4 ± 8.6	<0.0001
Inferior area	75.9 ± 32.5	137.6 ± 7.3	<0.0001
Nasal area	58.6 ± 19.4	78.2 ± 7.9	0.001

POAG = primary open-angle glaucoma; RNFL = retinal nerve fiber layer.

There was a statistically significant positive association between mf-VEP amplitude and RNFL thickness in the group of POAG eyes (r = 0.45, p = 0.016), whereas in control eyes RNFL and mf-VEP amplitude in Ring 1 were uncorrelated (r = −0.01, p = 0.985). Subsequently, there was a statistically significant positive correlation between average RNFL thickness and and average mf-VEP amplitude (r = 0.43, p = 0.021) in the POAG group, and no evidence for correlation in the control group (r = −0.03, p = 0.932).

## Discussion

Glaucoma is an optic neuropathy characterized by retinal ganglion cells death and corresponding nerve fiber layer loss, which results in characteristic visual field loss and causes blindness [1,2]. As a result, determining progression is crucial for effective clinical management of patients with glaucoma, while delay in recognizing progression may result in significant visual loss. Documented progression of glaucoma not only results in reevaluation of visual prognosis, but may also result in modification of treatment strategies. A number of approaches for identifying progression have been proposed by SAP. However, it is evident from the literature that no consensus exists regarding the best method for differentiating whether a visual field defect is stable or progressing. The problem is the high variability of SAP particularly in areas of visual field loss; the test-retest variability increases in areas with decreasing visual field sensitivity [12-15].

Recent research has suggested that variability of subjective tests results poses a significant problem in glaucoma diagnosis and follow-up. Hence objective tests like OCT and mfVEP have tried to improve reliability of glaucoma diagnosis and progression detection. In our study, our results demonstrated that in glaucomatous eyes the RRD of mfVEP in ring 1, 2 and 3 were significantly lower as compared to normal eyes. This decrease is higher in the central (ring 1) and paracentral zone (ring 2) and lower in ring 3. On the contrary, the implicit time was very similar in the two groups. Klistorner et al. and Rodarte et al. state also that there is relatively modest increase in latency in patients with glaucoma, while Parisi et al. suggest that glaucoma could have a major effect on the latency of the conventional pattern-reversal visual evoked potential (cVEP) [16-18]. Grippo et al., in an effort to solve this discrepancy, have studied the cVEP and mfVEP latencies in the same group of patients and they found only modest delays in the VEP response of eyes with glaucomatous damage [19]. However, there is little information regarding the use of mfVEP in monitoring progression. Only recently, Wangsupadilok et al. presented a method that may be useful in monitoring the progression of functional deficits in glaucoma using the mfVEP [10].

Concerning with RNFL thickness measurement, our data showed that in glaucomatous eyes the mean RNFL thickness in all areas is lower than normal. Nevertheless the decrease is slightly higher in the inferior and superior region. These changes of the inferior and superior quadrant of the RNFL correspond to the glaucomatous visual field defects detected with SAP. Furthermore, despite the normal visual acuity, the RNFL thickness of the temporal area which includes the macular bundle is decreased. This suggests that OCT changes may precede VA loss. According to Omodaka et al., if the RNFL thickness in the temporal area is close to 40 μm there is significant risk for decreasing of VA due to glaucoma [20].

## Conclusions

In conclusion, the principal message of our study is that, although SAP is the gold standard to evaluate glaucomatous neuropathy, the joint use of mfVEP and OCT could be useful in better monitoring glaucoma progression, thereby allowing more appropriate treatment of the disease.

## Competing interests

The authors declare that they have no competing interests.

## Authors’ contributions

MM conceived of the study, participated in its design, collected data and drafted the manuscript. GG conceived of the study, participated in the design of the study, collected data and revised critically the manuscript. IC performed the statistical analysis and helped drafting the manuscript. AR collected data and revised critically the manuscript. IL participated in the design of the study and revised critically the manuscript. All authors read and approved the final manuscript.

## Pre-publication history

The pre-publication history for this paper can be accessed here:

http://www.biomedcentral.com/1471-2415/12/34/prepub
